# Tools for simulating evolution of aligned genomic regions with integrated parameter estimation

**DOI:** 10.1186/gb-2008-9-10-r147

**Published:** 2008-10-08

**Authors:** Avinash Varadarajan, Robert K Bradley, Ian H Holmes

**Affiliations:** 1 Computer Science Division, University of California, Berkeley, CA 94720-1776, USA; 2 Biophysics Graduate Group, University of California, Berkeley, CA 94720-3200, USA; 3 Department of Bioengineering, University of California, Berkeley, CA 94720-1762, USA

## Abstract

Controlled simulations of genome evolution are useful for benchmarking tools. However, many simulators lack extensibility and cannot measure parameters directly from data. These issues are addressed by three new open-source programs: GSIMULATOR (for neutrally evolving DNA), SIMGRAM (for generic structured features) and SIMGENOME (for syntenic genome blocks). Each offers algorithms for parameter measurement and reconstruction of ancestral sequence. All three tools out-perform the leading neutral DNA simulator (DAWG) in benchmarks. The programs are available at .

## Abstract 2

Three tools for simulating genome evolution are presented: for neutrally evolving DNA, for phylogenetic context-free grammars and for richly structured syntenic blocks of genome sequence.

## Rationale

Almost every kind of genome annotation tool has, at some point in its lifecycle, been benchmarked on simulated data. Exceptions occur when the data are too poorly understood to be simulated, or so well understood that simulation is unnecessary. For the regime in between, generation of synthetic datasets is a nontrivial task. To accurately predict the strengths and weaknesses of a tool, one needs to generate simulated data with the same underlying statistics as the real data on which the tool will eventually be used. Relevant statistics include compositional bias (including local deviations from randomness like microsatellites or inverted repeats), phylogenetic correlations (including tree topology and branch lengths), hetergeneous substitution and indel rates, structured and conserved features (such as genes or binding sites) and context-dependence of mutational parameters. All the above factors lead to fluctuations in information density that can reduce the effectiveness of an annotation tool in practice.

A few concrete examples of applications that have been critically evaluated using simulated data are protein-coding genefinding [1], identification of transcription factor binding sites [2] and other functional non-coding DNA [3], phylogenetic reconstruction using distances based on substitutions [4,5] or indels [6], protein homology detection [7] and protein multiple alignment [8]. This list is a gross under-sampling from recent years, based on a citation search using the simulators described below; since simulation is almost a prerequisite for any new tool, the full list is much longer than this.

A number of tools address different aspects of the sequence simulation problem over long evolutionary timescales. Some of these tools are primarily protein-coding simulators; some handle DNA, and some do both. ROSE [9] is a canonical model for neutral sequence that implements substitution and indel events. DAWG [10] implements similar mutations to ROSE but is based on a deeper theoretical analysis of the underlying model, which is somewhat similar to the 'long indel' model of statistical alignment [11]. Several programs simulate substitution processes without indels; a recent example, which generates data of a given dinucleotide content (with a somewhat constrained choice of substitution model), is SISSIz [12]. Protein simulators include SimProt [13], Indel-Seq-Gen [14] and EvolveAGene [15]; of these, Indel-Seq-Gen offers the most realism, with rate heterogeneity and a diverse set of mutations. Simulators for genomic sequence (including conserved features) include EvolSimulator [16], which simulates gene family dynamics, and PSPE [17], which simulates turnover in promoter regions.

Many of these model-based simulation tools share a fundamental deficiency in their treatment of missing parameters and data. Specifically, they often lack robust functionality for: direct measurement of the model parameters from data; or integrated inference of missing information (gene boundaries, evolutionary histories, and so on) via the underlying model. The first aspect is important because the properties of the synthetic dataset may be parameter-dependent; the second aspect is important because even with direct measurement, the parameters measured may be strongly biased by the annotation of the training data.

Equally seriously, few of these models capture even a fraction of the true variety of genome features. At the mutational level, context-dependent substitution and indel rates (within which category we include microsatellite expansion and contraction) can have a significant impact on prediction accuracy [18]. At the level of selection for particular genomic features, a realistic simulator should model all the commonly encountered features of genomes, including protein-coding genes, non-coding RNA genes, conserved elements (such as binding sites), pseudogenes and transposons. All of these can bias different predictors in different ways.

There are notable exceptions to the above sweeping critique. DAWG does include a Perl script to estimate indel rates directly from data. SISSIz offers some heuristics for estimating parameters, without proof of the convergence or accuracy of these estimates. EvolSimulator does combine evolution of gene families and intergenic regions, but does not model the other genomic features mentioned above; nor does it provide tools to measure rate parameters. Indel-Seq-Gen is restricted to protein sequences; it offers domain-specific mutational parameters and includes events such as indels and domain shuffling but, again, does not offer a way to estimate these parameters. PSPE simulates promoter turnover, including loss-of-function mutations; the PSPE paper also describes ways to estimate parameters for promoter turnover directly from data, though it does not provide software to do this.

We have developed three related simulation engines: GSIMULATOR, SIMGRAM and SIMGENOME. The first, GSIMULATOR, models substitutions and indels in neutrally evolving DNA, with context-dependent rates for those events. The second, SIMGRAM, samples paths from a generic class of models called 'phylo-grammars', which include a broad class of models for proteins, coding DNA sequences, RNA genes, promoters and other features. The third, SIMGENOME, combines the previous tools with a rich diversity of genomic features (proteins, RNAs, pseudogenes, promoters and transposons) backed by a comprehensive repository of empirically measured substitution and indel rates, drawn from previously published analyses of large datasets such as PANDIT [19] and the 12 sequenced *Drosophila *genomes [20], and collected for the first time in a single place.

All three tools are based on generative models that offer efficient algorithms for parameter inference, annotation and/or ancestral reconstruction. The particular generative models used are transducers [21] and phylo-grammars [22], which both may be viewed as subsets of the set of mutational grammars. The theoretical advantages are several. One can learn parameters directly from data: for phylo-grammars we use the previously published XRATE program [22], while GSIMULATOR has a transducer estimator built-in. Generative grammars also offer parametric modularity: one can isolate important or repeated parameters, and place these conveniently at the top of the grammar file so that they can easily be edited or replaced with re-estimated parameters. Equally significantly, our grammars offer structural modularity. All the tools described here use documented file formats for representing their models. One can easily break down the grammar into sub-models, add new features or modify existing ones, without the need for directly modifying or re-compiling the underlying program code.

The present work is addressed at the problem of simulating syntenic blocks of genome sequence. We did not attempt to model genome rearrangements or duplications in this first release of our simulator, despite a clear need for benchmarking of genome rearrangement algorithms [23-30]. This was primarily because (at the time of writing) we lacked a unified framework for measurement in such models, or for dealing with missing data. In contrast, each simulation tool that we describe here is consistently complemented by robust measurement and reconstruction tools [22,31]. Adding rearrangement or duplication events would, however, be a natural future extension to our work. Another related but distinct area that we have omitted is evolution at the population level. Theoretical frameworks for populations exist, such as the coalescent [32] and super-processes [33], but exact inference in those frameworks is a significantly more challenging problem. Several recent simulators have addressed evolution at the shorter timescales associated with population genetics, incorporating effects like recombination, geographic migration, demography and ecology [34-36].

## Results

The fundamental component models of our simulator each have applications in training, simulation and inference. We propose that a single coherent evolutionary theory, encompassing and unifying these three aspects, is an extremely desirable feature for a simulation framework. There can be no simulation without parameters, which must be measured using some model; the parameters are typically measured from data that must have been aligned or annotated, using some model; and the alignment or annotation tools must be benchmarked using simulations, also using some model. The common feature of all the approaches described here, which we seek to emphasize, is that all three of these models are the same: parameter estimation, annotation and simulation can all be conducted using the same probabilistic model.

We now describe three tools. GSIMULATOR randomly generates alignments of neutrally evolving DNA; SIMGRAM randomly generates alignments of generically structured features under selection; and SIMGENOME combines these to randomly generate alignments of syntenic regions in genomes, using a reasonably detailed model of the genomic feature landscape. All three of these programs, together with examples of input/output files and command-line usage, may be accessed via the webpage [37].

### GSIMULATOR: a transducer-based simulator for neutrally evolving DNA

The GSIMULATOR tool simulates the neutral evolution of DNA on a phylogenetic tree. Along each branch, substitution and insertion-deletion mutations are modeled, using a context-dependent transducer. The theory of transducers is described in previous work [21] and summarized in the Materials and methods (see 'Sampling from lexicalized transducers'). Essentially, a transducer is a finite-state machine, similar to a Pair hidden Markov model ('Pair-HMM'), that mutates a sequence by introducing random substitutions and indels.

The GSIMULATOR transducer is context-dependent, meaning that the substitution and indel 'rates' are dependent on the past *K *absorbed and emitted symbols, where *K *is a parameter that can be configured. The purpose of allowing context-dependence is to model local sequence-dependent fluctuations in substitution and indel rates, such as methylation-dependent CpG deamination, microsatellite expansion and contraction, and 'micro-duplications' or 'micro-inversions' corresponding to *K *nucleotides or fewer.

GSIMULATOR also permits flexible modeling of gap length distributions, by allowing *N *multiple degenerate insertion and deletion states, where *N *is a configurable parameter. In the absence of context-dependence (that is, when *K *= 0), this yields a mixture of geometric distributions for the lengths of gaps. The distribution is more complicated for context-dependent transducers (*K *≥ 1), since the gap length depends on the inserted (or deleted) sequence.

As with all the methods described in this paper, GSIMULATOR is a trainable simulator, meaning that the parameters can be directly estimated from pairwise alignment data (and need not be 'guesstimated' by a user). Compositional biases of gene predictors, aligners, motif finders and other annotation tools can be exquisitely sensitive to the underlying evolutionary statistics, so this feature is extremely important for a robust simulator.

### SIMGRAM: a phylo-grammar simulation tool

The SIMGRAM tool generates sample alignments from a user-specified phylogenetic context-free grammar, or 'phylo-grammar'. In contrast to the transducers used by GSIMULATOR, the phylo-grammars of SIMGRAM can model genome features under finely structured selection, including co-ordination of the relative layout of these features (analogously to the way in which a human-language grammar specifies the layout of the various parts of speech). Examples of features that can be so modeled include protein-coding genes [1,18], non-coding RNA genes [22,38,39], protein binding sites [40], protein domains [41,42] and protein secondary structure [22,43]. The full theory of phylo-grammars has been widely described (see the above-cited applications by ourselves and others); a brief introduction can be found in the Materials and methods (see 'Sampling from phylo-grammars').

The phylo-grammar format used by SIMGRAM is the same as that of the XRATE program, a previously described tool that uses the Expectation Maximization algorithm to estimate the rate and probability parameters of any custom phylo-grammar [22]. Therefore, XRATE can be used to estimate simulation parameters directly from training data, then SIMGRAM can be used to generate synthetic data with similar properties, but no direct homology (excluding cases where the phylo-grammar itself encodes homology information [41,42]). This represents a new application of XRATE: the SIMGRAM program has not been previously described, and previous uses of XRATE have involved using the phylo-grammar to annotate sequence, or to measure substitution rates that are themselves of direct interest. (Thus, as with all the methods here, the generative model underpinning SIMGRAM can readily be trained on data and used for annotation and inference.)

An appealing feature of phylo-grammars for simulating an evolving and feature-rich genome sequence is that it is extremely easy to combine several sub-models into an all-encompassing model. Furthermore, the XRATE format allows several features that are useful for simulation. One such feature is parametric models, where the rates and probabilities are constrained to have a particular functional form that depends on a smaller parameter set. This is useful to construct models that have desired symmetry properties, such as strand-symmetric substitution processes, or *K*_*a*_/*K*_*s *_codon models. The XRATE format can also approximate context-dependent substitution rate models, using the technique of [44]. Finally, a powerful macro language exists that can be used to compactly describe grammars with many states, or to model lineage-specific parameterizations.

The DART (DNA, Amino Acid and RNA Tests) package, in which SIMGRAM is distributed, includes reproductions of several previously published phylo-grammars that can be simulated using SIMGRAM. Examples include models of secondary structure for proteins [43] and RNA [38], as well as a number of point substitution models for protein and nucleic acid sequences. The phylo-grammar format is described in full online [45].

### SIMGENOME: a feature-rich phylo-grammar for genome alignments

So far, we have described the GSIMULATOR program for context-dependent mutation of neutral DNA, and the SIMGRAM program for mutation of structured features under selection. Each model has its strengths and weaknesses: GSIMULATOR richly models neutral DNA, but not features under selection, whereas SIMGRAM has better models for such features but lacks GSIMULATOR's context-dependent rates or sophisticated indel model. We now describe a program that combines these approaches, using a modular framework that can easily be extended to incorporate future, specialized feature-simulators.

The combined simulator, SIMGENOME, starts by generating a multiple alignment from a template phylo-grammar that includes a rich array of genome features. The features are described below in more detail, together with outlines of how the template can be extended by an expert user to incorporate new features.

In the generated alignment, certain columns are flagged as intergenic. The SIMGENOME program then repeatedly calls GSIMULATOR to generate alignments of neutral DNA corresponding to these intergenic regions, and splices them into the main alignment. This process is extensible: the template phylo-grammar can be edited to add new features or change the underlying parameters of the model. Furthermore, other external feature simulators can be specified in the template grammar, and their output alignments will be spliced into the main alignment in exactly the same way as GSIMULATOR's output is.

The features modeled by the template phylo-grammar include protein-coding genes (with a rough approximation to exon-intron-untranslated region (UTR) structure that includes exon length distributions), non-coding RNA genes, conserved elements (such as transcription factor binding sites), pseudogenes and DNA transposons with terminal inverted repeats. Features can appear on forward or reverse strands. All features are annotated in the generated alignment, so that their recovery in automated benchmarks can be assessed.

The sub-models that generate these features use substitution rate parameters that all were estimated directly from the following experimental datasets, using the XRATE program (and could be re-estimated from alternative datasets).

#### The protein-coding gene model

The protein-coding gene model uses an empirical, fully reversible and otherwise unconstrained 61×61 rate matrix over codons, estimated in previous work [46]. Frame-preserving deletions are allowed. The training set for this model was the PANDIT database consisting of DNA-level alignments of protein domain families [19].

#### The non-coding RNA gene model

The non-coding RNA gene model treats gaps as a fifth character and therefore uses (4 + 1) × (4 + 1) single-stranded nucleotide and (4 + 1)^2 ^× (4 + 1)^2 ^double-stranded base-pair rate matrices that are fully reversible and otherwise unconstrained, and were estimated separately. The training set for this model consisted of alignments provided with the program CONSAN [47], which in turn were derived from the European large subunit rRNA database [48]. The initial probability distribution over base-pairs in double-stranded regions was also used to generate the terminal inverted repeats in simulated DNA transposons (although these features subsequently evolve under a neutral model, so that they do not display the compensatory mutations characteristic of non-coding RNA (ncRNA) genes under selection).

#### The strand-symmetric neutral substitution model

The strand-symmetric neutral substitution model that underpins the pseudogene and transposon models treats gaps as a fifth character and was trained on a random 1% of alignments of 12 *Drosophila *genomes [20,49]. The alignments themselves were made using the PECAN program [50]. The model was constrained to be strand-symmetric and reversible using XRATE's parameterization functionality. A slower, ungapped version of this substitution rate matrix is also used to model conserved features.

#### The transducer model

The transducer model used by GSIMULATOR to simulate intergenic sequence evolving under a neutral context-dependent model was trained on a set of pairwise alignments drawn from a subset of twelve-species *Drosophila *alignments, which were made using the PECAN program [50]. The subset was drawn from approximately 5% of the original multiple alignment data, to which a 95% minimum percentage identity threshold was applied.

#### The frequencies and length distributions

The frequencies and length distributions of genomic features were estimated from the *Drosophila *genome literature [20,51,52] using *Minos *as a model for DNA transposons [53,54].

The underlying template phylo-grammar is written using the publicly documented XRATE format and can be readily edited. High-level parameters, such as the frequencies with which genes or other features appear, are declared at the top level of the grammar and may be easily changed. We invite users to try adding sub-models representing new features of relevance to benchmarking (or to consult with us in this process). New sub-models can be parameterized directly from data using XRATE, and the parameters copied and pasted into SIMGENOME's grammar; this also applies to the existing models, which can be re-trained and/or re-parameterized (for example, to reflect differing codon usage or GC content). Even greater extensibility is afforded by the modular plug-in architecture that allows the use of third-party programs to generate features that cannot currently be simulated by SIMGENOME's phylo-grammars or transducers; such as tandem arrays, long-range duplications, and so on.

The following are examples of features that are not currently included in the SIMGENOME model, but are possible using SIMGRAM and could be incorporated by modifying SIMGENOME's grammar file.

#### Codon frequencies

The PANDIT dataset that was used to estimate the SIMGENOME codon model spans a wide spectrum of compositional and codon usage biases. In terms of overall patterns of conservation and suppressed mutation rates, we consider it an acceptable general model of codon substitution. For example, if one is benchmarking a motif-finding tool, the coding regions generated by SIMGENOME will result in more false positives than non-coding regions (because the level of conservation is higher) and this may be adequate for the purposes of that benchmark. However, for other purposes, one might conceivably want a richer parametric model that takes account of genome-specific effects such as compositional bias, codon bias, transition-transversion ratios or CpG methylation-induced deamination. Using such a parametric model is a simple case of swapping out the relevant rate matrix in the SIMGENOME grammar file. The model can be fit to data using XRATE in the normal way. We are preparing a manuscript describing a direct comparison between XRATE and PAML for these purposes, including Perl code for generating such richer parametric models (Heger A, Ponting C and Holmes IH, in preparation).

#### Lineage-specific parameterizations

The SIMGRAM macro language allows for different parameters on different branches of the tree. We have not made use of this feature in the SIMGENOME grammar, since its usage is somewhat dependent on the phylogenetic clade under investigation: one may (for example) want to use different parameters on every branch, on a single internal branch, or within a specific clade. With reference to the XRATE documentation, it is quite possible to design grammars that make use of this feature, so that the model may (for example) use different codon frequencies or compositional biases in different parts of the tree.

#### Loss-of-function mutations

The SIMGRAM format also allows for lineage-specific evolution of whole features, in the manner of the DLESS program by Pollard *et al. *[55]. Internally, we have developed phylo-grammars modeling loss-of-function mutations in protein-coding genes, for investigations of pseudogene evolution. We have not included loss-of-function mutations in this first release of SIMGENOME, but it would be quite feasible to extend it without needing to write new code.

#### Splice sites, initial methionines, UTRs and other aspects of protein-coding gene structure

The grammar of SIMGENOME currently includes a crude semblance of exon and intron structure, in order to reproduce the broad compositional fluctuations associated with protein-coding genes. This currently includes slow-evolving sequence at the exon-intron boundaries, as a mock-up of splice site conservation. The current release does not, however, model protein-coding genes at a sufficient level of detail to be used as positive examples for a protein-coding gene predictor. One could readily modify the simulator to do this, modeling splice sites as GT-AG and perhaps even AT-AC donor-acceptor pairs, and including other features such as poly-A signals, initial ATGs, TATA boxes and UTRs. These sorts of feature are straightforward to add to a phylo-grammar framework.

#### Higher-order correlations between codons

The SIMGRAM distribution includes example phylo-HMMs demonstrating higher-order correlations between amino acids in protein alignments. For example, there is a replica of the Thorne-Goldman-Jones 3-state phylo-HMM for modeling secondary structure [43]. Given a suitable parameterization to map from amino-acid substitution rate matrices to codon matrices, it would be straightforward to use something like this to model higher-level correlations between codons in SIMGENOME's coding-DNA regions. One may reasonably ask why we have not included such higher-order dependencies in the first release of SIMGENOME, when we have included higher-order context-dependencies in the intergenic regions. The answer is that intergenic features (such as microsatellites or conserved regions) may include strong compositional biases extending over tens of bases and so generally contribute more to fluctuations in information density than correlations between codons, which are typically weak, local and often detectable only at the level of the encoded amino acids [43].

#### Detailed length distributions

Most of the length distributions of features modeled in SIMGENOME are geometric (the simplest kind of distribution that one can model with an HMM). By chaining together duplicate states, it is possible to generate more complex (realistic) length distributions. For example, the SIMGENOME grammar file includes functions for generating a peaked 'negative binomial' distribution (also known as the 'Pascal' distribution) for exon lengths; so there are examples that can be used to do this. However, a straightforward geometric distribution was empirically found to be a better fit to exon length data in *Drosophila*.

### Evaluation: benchmarking a non-coding RNA predictor

Evaluating a simulation tool is a slightly different problem from evaluating an annotation tool, such as a gene predictor. When benchmarking a predictor, one is typically interested in minimizing the number of false positives that the predictor finds in a null dataset at a given threshold of the prediction score cutoff. One can eliminate all false positives by setting the score threshold arbitrarily high, but at the cost of also missing all the real genes in the dataset. To get a true picture of the predictor's performance, one must therefore consider how the number of false positives varies as a function of this threshold; or (more meaningfully) as a function of the sensitivity of the predictor, that is, the number of real genes that it correctly detects at a particular score threshold.

A common practice is to use a set of real genes to evaluate the sensitivity of the predictor, but to use simulated null data to evaluate the false positive rate. The reason for using simulated null data, instead of a real DNA sequence that does not contain any genes, is the paucity of negative annotations: it is difficult to demonstrate experimentally that a particular sequence of DNA definitively does not contain any genes. This is particularly true of hard-to-identify genes, such as RNA genes or short open reading frames.

A good simulator, therefore, is one that closely reproduces the statistics of real DNA, and generates a similar number of false positives to real DNA. In practice, the second criterion rests on the choice of statistics for the first criterion In general, if the statistical model is too simplistic (for example, omitting low-complexity regions), then the complexity of the simulated DNA will be more than it should be, leading to fewer false positives. To take a specific example: a DNA simulator that randomly emits a sequence of independent, identically distributed symbols will match the nucleotide-level composition of real DNA, but will not reproduce the short-range fluctuations in information content that can be found in real DNA, and so will generate fewer false positives in a motif-finding benchmark than a simulator that includes short-range complexity fluctuation phenomena (such as microsatellites).

On this basis, we argue that a measure of a good probabilistic simulator is that its model (after being fit to real DNA) should maximize the number of false positives for a given predictor at a given sensitivity. Another way of putting this is that the simulator should provide the most stringent possible benchmark for the gene predictor, by minimizing the area under the receiver operating characteristic (ROC) curve over the range of interest (see below).

To evaluate the simulation engines described here, we used them to estimate the false positive rate (FPR) for a computational whole-genome screen for conserved structural RNA genes, conducted using XRATE [22]. We chose RNA gene prediction as a test case because it has an extremely high FPR, whose actual extent is still unknown [39,56,57]; and because the estimated FPR for this screen is highly sensitive to the underlying properties of the simulation engine, making it a good motivator of increased realism [12,58].

Specifically, the FPR was estimated by sliding a window across the simulated alignments, and running XRATE on each window using an RNA gene-prediction grammar. This grammar models the distinct patterns of nucleotide substitution in RNA genes, including covariation of base-paired nucleotides, and is closely related to the EVOFOLD program for comparative RNA gene prediction [39] and the PFOLD program for comparative RNA folding [59]. The grammar itself, along with detailed instructions for reproducing the screen, can be found online [37]. The full rationale underlying the development of the grammar, and its critical evaluation (as a genefinder) and comparison to related grammars, will be described elsewhere (Bradley RK, Uzilov AV, Skinner M, Bendaña YR, Barquist L and Holmes I, submitted).

The plots show ROC curves where the FPR is plotted against the sensitivity of the screen (as measured using annotations of known ncRNAs in *Drosophila melanogaster*), as a parametric function of the score threshold for the screen. Since ncRNAs represent positive results for this screen, and we already have a set of known curated ncRNAs for *D. melanogaster*, we omitted the ncRNA submodel from the SIMGENOME grammar for these tests.

**Figure 1:**
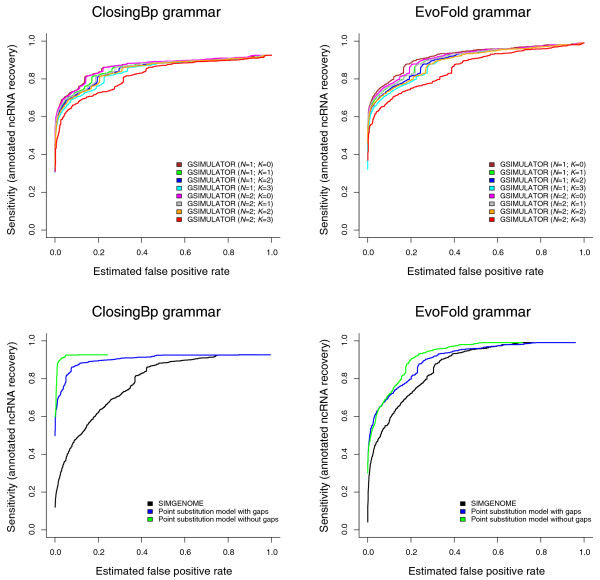
Receiver operating characteristic (ROC) curves for two non-coding RNA prediction algorithms, ClosingBp (Bradley RK, Uzilov AV, Skinner M, Bendaña YR, Barquist L and Holmes I, submitted) and EVOFOLD [39] (implemented using XRATE), using GSIMULATOR and SIMGENOME models to estimate the false positive discovery rate. These curves illustrate the general principle that the more realistic a simulation model, the higher the estimated false positive rate (FPR). This trend is independent of the gene-prediction algorithm used. The upper panes show results for GSIMULATOR: it is seen that more complex indel length distributions (*N*) and, in particular, context-dependence (*K*) both increase the FPR. The lower panes show results for SIMGENOME and component models, where the FPR is increased by including gaps (which amplify fluctuations in information content, due to their typically being treated as 'missing information') and genomic features (some of which evolve at a slower rate than neutral sequence). The reason that the asymptotic sensitivity is less than 1.0 is that our benchmark used a sliding-window approach, predicting at most one non-coding RNA (ncRNA) in each window. Our set of real ncRNAs was taken from multi-genome *Drosophila *alignments produced by the PECAN program [50]; in each case, to ensure a fair comparison, we took a window of the PECAN alignment surrounding the annotated ncRNA, with the size of this window matching the size of the sliding-window that was used on the simulated null data. Some of the positive ncRNAs in these PECAN-aligned windows score so poorly under the gene prediction model - for example, due to inaccuracies in the PECAN alignment of that window - that the predicted ncRNA is consistently placed in the wrong location within the window. These real ncRNAs are, therefore, never detected, no matter how low the scoring threshold, setting an upper limit on the achievable sensitivity.

The results are shown in Figure 1. The general conclusion is that increased realism makes for a higher FPR. In the case of GSIMULATOR, increasing either *N *or *K *radically increases the FPR; in the case of SIMGENOME, inclusion of conserved genomic features with slower evolutionary rates markedly increases the FPR relative to both a pure point substitution model as well as the most realistic GSIMULATOR model.

We also compared our simulation methods, GSIMULATOR and SIMGENOME, to DAWG [10], a widely cited program for simulation of neutral substitution and indel events. We chose DAWG because it most closely exemplifies the goals we have identified here: it is clearly based on an underlying evolutionary model and provides tools for estimating the parameters of the indel model directly from sequence data. It appears to be the leading general-purpose simulator at the time of writing. Other simulators (such as PSPE) are richer, but do not provide the parameter-estimation functionality that DAWG does.

The parameters for DAWG were as follows. We used the same general-time reversible substitution model (REV) that we estimated from PECAN alignments of *Drosophila *genomes. DAWG's 'geometric' indel model (that is, geometrically distributed indel lengths) was parameterized using the script provided with the program. Although the 'power-law' model for indel lengths gave a better fit, it produced alignments that were mostly gaps. DAWG allows for heterogeneous rates and invariant sites using the Γ + *I *model for rate heterogeneity [60], also providing some example parameters for this model (γ = 1, ι = 0.1), which we used for these simulations.

**Figure 2:**
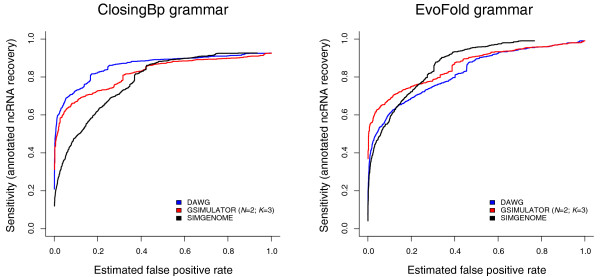
ROC curves for two non-coding RNA predictors, ClosingBp (Bradley RK, Uzilov AV, Skinner M, Bendaña YR, Barquist L and Holmes I, submitted) and EVOFOLD [39] (implemented using XRATE), comparing DAWG [10] to the richest GSIMULATOR and SIMGENOME models. The three curves for each gene predictor clearly illustrate that increased model richness (DAWG → GSIMULATOR → SIMGENOME) yields higher estimated FPR. See the caption to Figure 1 for an explanation of why the asymptotic sensitivity is less than 1.0.

Figure 2 compares DAWG with the most realistic GSIMULATOR and richest SIMGENOME models. The figure shows that the context-dependence modeled by GSIMULATOR and the genomic features modeled by SIMGENOME result in much tighter false-positive estimates than DAWG produced.

Since DAWG's example γ and ι parameters (which determine the rate heterogeneity and conserved-site density) were manually adjusted for human-chimp alignments by the program's author, they may be an underestimate for *Drosophila *(where conserved elements are more closely spaced than in primates, due to smaller genomes and a higher deletion rate of nonfunctional DNA). However, there is no automated method for setting these parameters in DAWG; nor does the DAWG package or paper provide explicit guidance on how to relate these parameters to straightforward statistics on genomic feature density. (The SIMGENOME grammar file, in contrast, includes comments that outline the derivation of the feature distribution parameters from published *Drosophila *annotations.) Further, the GSIMULATOR program includes a fully automated training procedure, has no model for conserved sites or rate heterogeneity (other than context-dependent substitution) and nevertheless generates a higher false prediction rate than DAWG, even when these features are enabled in DAWG (Figure 2). Since SIMGENOME, in turn, generates a higher FPR than GSIMULATOR (Figure 1), we reason transitively that SIMGENOME is also a more realistic simulator than DAWG. Correspondingly, we note that this argument strongly motivates an automated tool (or even a simple heuristic) for estimating the γ and ι parameters of DAWG's heterogeneous-rate model. There is considerable analysis of such models [60], so this may be a reasonable goal of future study.

## Discussion

We have described an extensible system for genome simulation that includes a rich array of features and parameters, together with integrated methods for estimating those parameters, and comes with a comprehensive repository of mutation rate measurements for genomic features and intergenic DNA. Features modeled by our simulator include protein-coding and non-coding RNA genes, pseudogenes, transposons, conserved elements, microsatellite expansion/contraction and other context-sensitive substitutions and indels in neutrally evolving regions.

There are several obvious extensions to this work. We did not include large duplications or rearrangements in our simulator because, at the time of writing, we lacked up-to-date methods for measuring the frequencies of these events in evolution (although see [61]). However, such events would be an obvious extra layer to add to our system. A variant on this would be to simulate gene family dynamics using a birth-death model [62,63] for benchmarking of orthology/paralogy prediction methods. This might also be achieved by constructing gene features from explicit models of protein domains, for example, based on HMMs from the PFAM database. Likewise, we could generate ncRNA features based on explicit families (such as tRNAs).

The power of efficient generative probabilistic models is that they provide a common framework for measuring parameters, simulating from those parameters, and reconstructing missing information such as ancestral genotypes or gene boundaries. Another strong advantage, which we have used for this work, is the ease with which such models can be extended to incorporate new features or variations on existing features. The technologies that we have used to build our simulator (phylo-grammars and transducers) can, in fact, be viewed as overlapping subsets of a larger set of mutational grammars. With the inevitable trend of richer prediction tools whose dependence on the intricate structure of sequence evolution becomes ever harder to predict, the future of such generative grammatical models is as bright in computational biology as it is in linguistics.

## Materials and methods

### Sampling from phylo-grammars

A phylo-grammar is a stochastic context-free grammar whose every state generates alignment columns (or groups of alignment columns). The residues in each row of such a column are related by an underlying phylogenetic tree (whose topology remains constant), using a continuous-time Markov chain subsitution model (which is allowed to vary from state to state). Each state in the grammar is allowed to generate several columns simultaneously; these sites then evolve co-ordinately, so that a state can (for example) emit three columns that evolve together as a codon triplet, or two distantly separated columns that evolve as a base-pair in a non-coding RNA alignment.

One can vary the evolutionary rate throughout the tree, and for this behavior to be state-dependent, so that different states in the grammar can have trees with different branch lengths. However, SIMGRAM requires that the tree topology stay constant throughout the alignment. (While it is mathematically straightforward to construct phylo-grammars with alternative tree topologies in the various states, such models are not implemented by SIMGRAM.)

Sampling from the phylo-grammar proceeds in two stages. First, a parse tree is sampled by repeated application of randomized grammar rules. Next, a Gillespie algorithm is used to simulate continuous-time Markov chain trajectories for each set of co-evolving columns [64].

### Sampling from lexicalized transducers

A transducer is a finite state machine, similar to a 'Pair-HMM', but conditionally normalized; thus, instead of emitting two sequences (as does a Pair-HMM), the transducer absorbs one sequence (*X*) as an input and emits the second sequence (*Y*) as an output. Practically, a transducer looks very similar to a Pair-HMM: it has match, delete and insert states, and the training and alignment algorithms are almost identical. The key difference is the probabilistic normalization: a transducer's emission and transition probabilities are normalized such that the likelihood of any state path π corresponds to the conditional probability *P*(π,*Y*|*X*), whereas in a Pair-HMM the state path likelihood corresponds to the joint probability *P*(π,*X*,*Y*). Conceptually, a transducer absorbs a symbol on the input, then decides (randomly) what state to go into, then emits a symbol (or symbols) on the output; in contrast, a Pair-HMM first changes state, then emits symbol(s) on either or both outputs.

A transducer models random changes in a sequence, as might occur on an individual branch of a phylogenetic tree. It is also convenient to introduce a special type of transducer, located at the root of the tree, that only emits sequence (that is, it has only 'insert' states, and lacks 'match' or 'delete' states to absorb an input sequence). Such a transducer is referred to as a 'singleton' transducer and is exactly equivalent to a single sequence-emitting HMM. The composition of an (emitting) singleton transducer with an (absorbing-and-emitting) branch transducer yields a machine that is exactly equivalent to a Pair-HMM.

A lexicalized transducer is one in which the probability parameters for making transitions between states, and for absorbing or emitting various symbols within states, are dependent on the last few symbols that were emitted or absorbed. This dependence allows the root transducer to generate higher-order compositional statistics of DNA (such as dinucleotide or trinucleotide frequencies). It also allows the branch transducers to model phenomena such as increased rates of indels and substitutions in low-complexity regions, such as microsatellites.

The lexicalized transducer underlying GSIMULATOR is a discrete mutator that introduces substitutions and indels at a frequency corresponding to a finite span τ of evolutionary time, corresponding (typically) to a short branch on a phylogenetic tree. For example, the pre-trained transducers that are distributed with GSIMULATOR, and parameterized from *Drosophila *alignments, correspond to a branch length of τ ≈ 0.07 substitutions per site. Longer branches of the tree, with length *T *> τ, are simulated by iterated application of the transducer (for example, a branch of length *T *= 0.35 corresponds to five repeated applications of an evolutionary transducer with branch length τ ≈ 0.07); branches of length *T *< τ are rounded up (if *T *≥ τ/2) or down (if *T *< τ/2).

In other words, evolutionary time *T *is treated as a discrete parameter, corresponding to some integer multiple of τ. This contrasts with many point substitution models that are used in molecular evolution (and in previous evolutionary simulators), which are based on continuous-time Markov chains. This behavior is deliberate and justified on the grounds of realism, as follows: it is extremely difficult to obtain closed-form matrix exponential solutions to the transition probabilities for context-dependent substitution and indel models of the kind used by GSIMULATOR. Indeed, only approximate solutions currently exist for context-dependent substitution models [44,65], and there is currently no good theory at all for context-dependent indels. We argue that the increased realism of context-dependence is easily worth a small reduction in granularity of the evolutionary time parameter. In cases where fine-grained time distinctions are critical, the SIMGRAM phylo-grammar simulator described here can be used instead.

The training procedure for GSIMULATOR is straightforward. The algorithm for estimating the parameters is essentially the same as the Baum-Welch algorithm for training a Pair-HMM from a pairwise sequence alignment [66]. The corresponding inference algorithms are not detailed here, but there exist a range of dynamic programming algorithms, from exhaustive inference to Markov Chain Monte Carlo algorithms [31,67-69] that can be used to impute alignments and evolutionary histories, to reconstruct ancestral sequences, or to annotate sequences in alignment-free (as well as alignment-dependent) ways. The particular algorithm used by GSIMULATOR to infer parameters is a variant on the Baum-Welch algorithm [66]. During training, GSIMULATOR finds the branch length τ from the underlying data using a Jukes-Cantor distance estimate [70].

For the four symbols of the DNA alphabet, order-*K *context-dependence multiplies the number of transducer parameters by a factor of 4^2*K*^. In practice, the quantity of training data (and potentially runtime and/or memory considerations) will limit the value of *K*. We find that a value of *K *= 3 yields a transducer that can reasonably be trained from eukaryotic genome alignments of the approximate size of *Drosophila*, but that this transducer takes a long time to train (especially if the degeneracy *N *of the indel states is greater than 1).

We do not currently have a specialized ancestral reconstruction tool for lexicalized transducers; instead, we have a more general tool. The previously published Handel program [68] is a Gibbs sampler for multiple alignments and ancestral sequence reconstructions under the TKF91 model [71]. More recently, it has been extended to model affine indel-length distributions (unpublished). Handel can also be coupled to any external program that implements a probabilistic scoring function for alignments; that is, given any ancestral reconstruction *A *in Stockholm alignment format [72], the program must compute and then output *P*(*A*,*S*|*M*) for some model *M*, where *S *represents the observed sequences at leaf nodes. (GSIMULATOR is itself one such program.) Handel then accepts the Markov Chain Monte Carlo move from *A *→ *A' *with probability:

$$\min\left(1,\frac{P(A^{\prime },S|M)P(A,S|T^{\prime })}{P(A,S|M)P(A^{\prime },S|T)}\right)$$

where *T *represents the TKF91 model; this is a Hastings ratio [73]. Asymptotically, this yields a direct sample from *P*(*A*|*S*,*M*).

### Generative model features

Most features of the SIMGENOME phylo-grammar are self-explanatory, either from this paper or by inspection of the commented grammar file (located in the subdirectory dart/grammars/SIMGENOME.eg). The length distribution of exons is a negative binomial distribution, or Pascal distribution, obtained by serial chaining of left-regular states, each of which emits a geometrically distributed number of codons. (The Pascal distribution is that of the sum of independent, geometrically distributed random variables.) This results in far fewer unrealistically shorter exons than would be generated by a geometric distribution, while allowing easy parameterization of the mean exon length.

The default GSIMULATOR model used by SIMGENOME has two insert states and two delete states, and uses three nucleotides of both input and output context. Roughly speaking, this corresponds to a mixture of geometric distributions over gap lengths; however, due to the input and output context, the precise length of inserted or deleted sequences will also depend on the sequence content of those indels.

## Abbreviations

DART, DNA, Amino Acid and RNA Tests; FPR, false positive rate; HMM, hidden Markov model; ncRNA, non-coding RNA; ROC, receiver operating characteristic; UTR, untranslated region.

## Authors' contributions

AV wrote GSIMULATOR. RB wrote the SIMGENOME phylo-grammar, ran the gene-prediction benchmarks and prepared the ROC curve figures. IH wrote SIMGRAM (and related programs), the SIMGENOME Perl script, and the manuscript. All authors read and approved the final manuscript.
